# Evaluating and implementing block jackknife resampling Mendelian randomization to mitigate bias induced by overlapping samples

**DOI:** 10.1093/hmg/ddac186

**Published:** 2022-08-06

**Authors:** Si Fang, Gibran Hemani, Tom G Richardson, Tom R Gaunt, George Davey Smith

**Affiliations:** Population Health Sciences, Bristol Medical School, University of Bristol, Bristol BS8 2BN, UK; Medical Research Council (MRC) Integrative Epidemiology Unit (IEU), University of Bristol, Bristol BS8 2BN, UK; Population Health Sciences, Bristol Medical School, University of Bristol, Bristol BS8 2BN, UK; Medical Research Council (MRC) Integrative Epidemiology Unit (IEU), University of Bristol, Bristol BS8 2BN, UK; Population Health Sciences, Bristol Medical School, University of Bristol, Bristol BS8 2BN, UK; Medical Research Council (MRC) Integrative Epidemiology Unit (IEU), University of Bristol, Bristol BS8 2BN, UK; Novo Nordisk Research Centre, Headington, Oxford OX3 7FZ, UK; Population Health Sciences, Bristol Medical School, University of Bristol, Bristol BS8 2BN, UK; Medical Research Council (MRC) Integrative Epidemiology Unit (IEU), University of Bristol, Bristol BS8 2BN, UK; Population Health Sciences, Bristol Medical School, University of Bristol, Bristol BS8 2BN, UK; Medical Research Council (MRC) Integrative Epidemiology Unit (IEU), University of Bristol, Bristol BS8 2BN, UK

## Abstract

Participant overlap can induce overfitting bias into Mendelian randomization (MR) and polygenic risk score (PRS) studies. Here, we evaluated a block jackknife resampling framework for genome-wide association studies (GWAS) and PRS construction to mitigate overfitting bias in MR analyses and implemented this study design in a causal inference setting using data from the UK Biobank. We simulated PRS and MR under three scenarios: (1) using weighted SNP estimates from an external GWAS, (2) using weighted SNP estimates from an overlapping GWAS sample and (3) using a block jackknife resampling framework. Based on a *P*-value threshold to derive genetic instruments for MR studies (*P* < 5 × 10^−8^) and a 10% variance in the exposure explained by all SNPs, block-jackknifing PRS did not suffer from overfitting bias (mean *R*^2^ = 0.034) compared with the externally weighted PRS (mean *R*^2^ = 0.040). In contrast, genetic instruments derived from overlapping samples explained a higher variance (mean *R*^2^ = 0.048) compared with the externally derived score. Overfitting became considerably more severe when using a more liberal *P*-value threshold to construct PRS (e.g. *P* < 0.05, overlapping sample PRS mean *R*^2^ = 0.103, externally weighted PRS mean *R*^2^ = 0.086), whereas estimates using jackknife score remained robust to overfitting (mean *R*^2^ = 0.084). Using block jackknife resampling MR in an applied analysis, we examined the effects of body mass index on circulating biomarkers which provided comparable estimates to an externally weighted instrument, whereas the overfitted scores typically provided narrower confidence intervals. Furthermore, we extended this framework into sex-stratified, multivariate and bidirectional settings to investigate the effect of childhood body size on adult testosterone levels.

## Introduction

Genome-wide association studies (GWAS) studies have discovered thousands of genetic variants that robustly associate with many different complex traits and disease endpoints in the past two decades. These findings not only lead to potential translatable opportunities for pharmaceutical target development and highlight biological mechanisms and pathways, but also facilitate endeavours in disease prediction and risk stratification using polygenic risk scores (PRS) (1). Trait-associated genetic variants can also be used as proxies for lifestyle risk factors in Mendelian randomization (MR) studies. Such an approach harnesses genetic data to strengthen causal inference in epidemiological research, and can be implemented in instrumental variables (IV) analyses (2–4).

MR studies rely on the selection of valid genetic IVs, which are robustly associated with the exposure of interest, affect the outcome only through the exposure being analyzed and which do not share a common cause with the outcome (5). Genetic IVs are conventionally selected from an independent dataset whose sample does not overlap with the dataset being analyzed using MR analysis, as overfitting bias may arise owing to the use of overlapping samples (6). This can often be challenging however, as GWAS are increasingly being performed by meta-analyzing several biobanks to achieve the maximum power to detect variants with smaller effects. Furthermore, when investigating exposures and outcomes which only a single biobank has measured in sufficiently large samples, avoiding participant overlap requires splitting the study population into subgroups that can limit statistical power (7). These issues could be avoided using the block jackknife resampling MR, an approach for causal inference in a single sample without participant overlap.

Jackknife resampling, also referred to a leave-one-out or N-fold cross-validation, is a form of instrumental variable derivation firstly described by Angrist and colleagues to obtain the fitted value during the first stage of two-stage least squares (2SLS) regression (8). This method can be applied to mitigate the bias in 2SLS conducted in finite samples when the instruments are weak, namely finite-sample bias or weak instrument bias (6,8). The application of block jackknife approach in MR was first proposed by Burgess and Thompson (9) as an approach to avoid a reduction in statistical power, while there are limited sources of data. Different from Angrist *et al.*’s jackknife approach, block jackknife resampling MR applies the jackknife resampling design in blocks to identify genetic instruments robustly associated with the exposure before performing 2SLS to ensure independence between the discovery and applied dataset (10,11). As such, this approach mitigates the residual correlation between the first and second stage of 2SLS and is conceptually similar to the cross-fitting strategy for instrumental variable regression which has been integrated with machine learning in econometrics research (11,12). Moreover, allele scores, or PRS, as a weighted sum of genetic instruments were also constructed in this method to mitigate weak instrument bias in MR using individual level data (9).

In this study, we have evaluated the use of block jackknife resampling to maximize sample sizes for both IV identification and MR analysis using simulated datasets (8). We compared this study design with a one-sample MR design using genetic instruments that were either identified in external GWAS or identified by GWAS on fully overlapping samples. To explore the optimal scenarios for this method, we also carried out a range of simulated MR analyses using allele scores constructed from external GWAS with a range of sample sizes. Next, we evaluated the three methods (IV selection using block jackknife resampling, external GWAS or overlapping GWAS) using real data by analyzing body mass index (BMI) data in the UK Biobank (UKB) and the largest BMI GWAS meta-analysis with no reported sample overlap with the UKB cohort. Finally, by applying the block jackknife resampling MR method, we investigated the sex differences in the genetically predicted effect of childhood body size on adult testosterone levels, where both sex-stratified measures were available on the large sample available in UKB. The influence of childhood body size on adult testosterone levels was then investigated in terms of direct and indirect effects after accounting for adult body size, by extending the jackknifing study design into a multivariable setting.

## Results

### Simulation analyses

#### Bias brought by overlapping samples in simulated data

**Figure 1:**
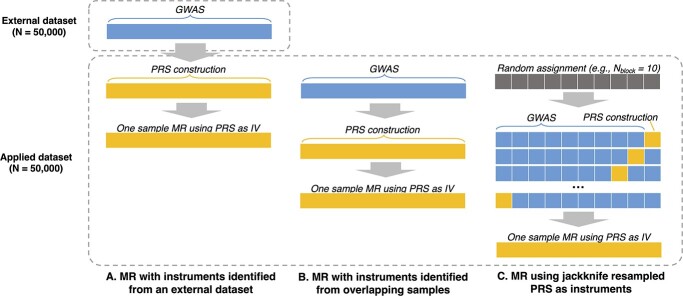
Schematic diagram showing the three frameworks for selecting samples for conducting GWAS and PRS construction in the simulated analyses. The three frameworks applied in the simulation analyses are (A) MR with instruments identified from an external dataset: SNPs and their weights used for PRS construction were identified from a GWAS on an external dataset, different from the applied dataset which is used for PRS construction and MR; (B) MR with instruments identified from overlapping samples: the same dataset is used for effect estimate, PRS construction and MR; (C) MR using jackknife resampled PRS as instruments: effect estimate, PRS construction and MR under the block jackknife resampling framework. Blue: dataset used to estimate SNP effects; Yellow: dataset used to construct PRS and carry out one-sample MR.

To investigate the effect of sample overlap and the advantages of using a block jackknife resampling framework to mitigate overfitting bias, we performed extensive simulations to construct PRS under the combination of three GWAS frameworks (block jackknife resampling, overlapping sample GWAS or external GWAS) and 13 different *P*-value thresholds. Externally weighted PRS was constructed based on an external GWAS (discovery dataset) with no sample overlap with the applied dataset. Overlapping sample PRS was constructed with 100% sample overlap between the discovery dataset and applied dataset. In block jackknife resampling, samples in the applied dataset were split into N_block_ blocks for SNPs selection as well as PRS construction. After generating the PRS for every block based on the GWAS performed on N_block_-1 blocks, we then combine samples from every block and use their PRS as the instrumental variable for the exposure in a 2SLS regression analysis using the full sample. Figure 1 shows a schematic illustration of the three approaches.

**Figure 2:**
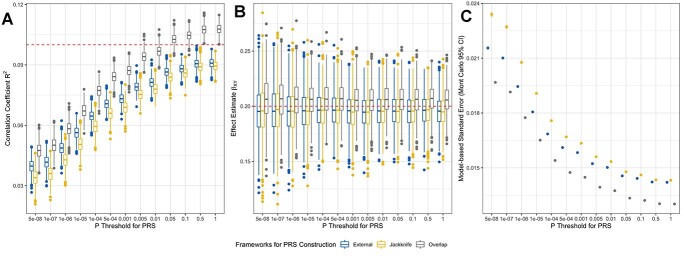
Results from simulation analyses comparing three methods for GWAS and PRS construction for prediction and MR. The performance of PRS constructed under three GWAS frameworks in phenotype prediction (**A**) and one-sample MR (**B** and **C**). (A) Boxplots showing the distribution of the correlation between simulated PRS and exposure. (B) Boxplots showing the distribution of the MR estimates (beta coefficients) of effect of the exposure on the outcome. (C) Scatter plot showing the model-based standard errors of the effect estimates in plot B and their Mont Carlo 95% CI. Red lines represent the parameters used in data simulation, i.e. the true value of correlation coefficient *R*^2^ (0.10) in plot A and the true effect from the exposure to the outcome ${\beta}_{XY}$ (0.20) in plot B.

An overview of the performance of simulated PRS is presented in Figure 2. Adjusted correlation coefficient $\hat{R}^2$ between PRS and the exposure phenotype represents the predictive ability of the PRS (Fig. 2A). Using a conventional *P*-value threshold to derive genetic instruments for MR studies (i.e. *P* < 5 × 10^−8^), our block jackknife resampling PRS did not appear to suffer from overfitting bias (mean $\hat{R}^2=0.034$; $bias\ \big[ Mont\ Carlo\ standard\ error, MCse\big]=-0.066\big[1.316\times{10}^{-4}\big]$) in comparison to the externally weighted PRS (mean $\hat{R}^2=0.040$; $bias\big[ MCse\big]=-0.060\big[1.105\times{10}^{-4}\big]$). However, genetic instruments derived from overlapping samples typically explained a higher proportion of variance (mean $\hat{R}^2=0.048$, $bias\big[ MCse\big]=-0.052\big[1.264\times{10}^{-4}\big]$) compared with the externally derived score. The detrimental impact of overfitting bias became considerably larger when using a more liberal *P*-value threshold to construct PRS (e.g. when $P=0.05$, mean $\hat{R}^2=0.103$, $bias\big[ MCse\big]=0.003\big[8.247\times{10}^{-5}\big]$), whereas block jackknife resampled estimates remained robust to overfitting (when $P=0.05$, mean $\hat{R}^2=0.089$, $bias\big[ MCse\big]=-0.011\big[8.400\times{10}^{-5}\big]$).

**Figure 3:**
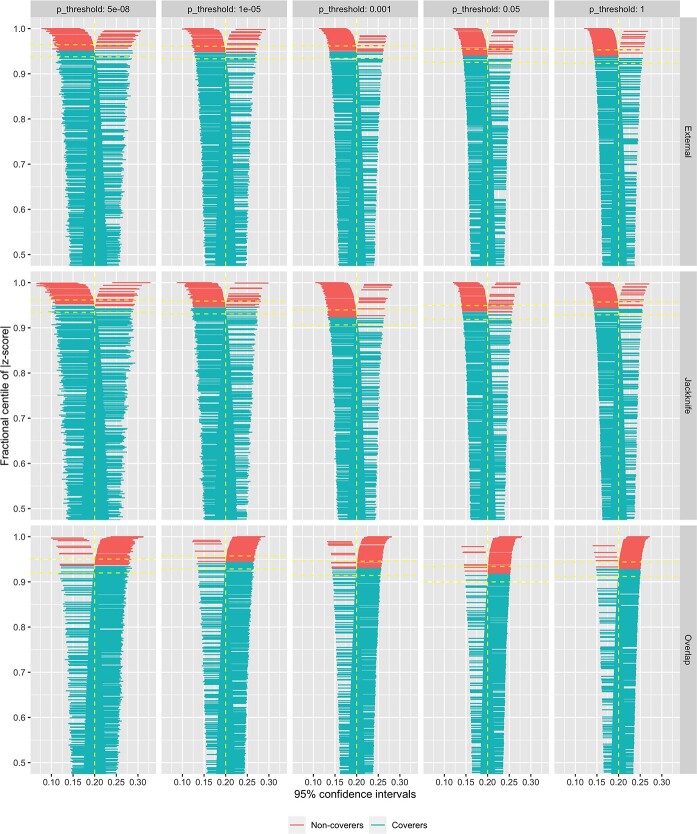
Zipper plots showing the 95% CIs and their coverage rates of a subset of the effect estimate generated in the primary simulation analysis. This plot shows the results from simulation analysis under 15 combination of different *P*-value thresholds (5 × 10^−8^, 1 × 10^−5^, 0.001, 0.05, 1) and method of PRS construction (external: externally weighted PRS; jackknife: block jackknife resampled PRS; overlap: overlapping sample PRS). Each plot shows the 95% CIs of the 50% of simulation results (i.e. 500), fractional-centile-ranked based on the absolute value of Z scores, which is defined as the $\big({\hat{\beta}}_{XY}-{\beta}_{XY}\big)/\hat{SE}\big({\hat{\beta}}_{XY}\big)$.

In one-sample MR, exposures instrumented by PRS constructed with overlapping samples showed slightly inflated effects on the outcome under all *P*-value thresholds (overall mean $\hat{\beta}_{XY}=0.206$, overall $bias=0.006$), whereas both block jackknife resampled PRS and externally weighted PRS suggested that effects slightly biased towards the null (both overall mean $\hat{\beta}_{XY}=0.196$; overall $bias=-0.004$) (Fig. 2B). Moreover, results derived using overlapping sample PRS had lower model-based standard errors for the effect estimate, whereas block jackknife resampled PRS produced results with higher standard error compared with externally weighted scores (Fig. 2C). Additionally, the coverage rates of nominal 95% confidence intervals (95% CIs) of the effect estimates are the lowest among results generated using the overlapping sample PRS (mean coverage = 92.9%), whereas MR estimates using externally derived PRS and jackknife resampled PRS have higher coverages, with a mean of 94.3% and 94.1%, respectively. The Monte Carlo standard error of the coverage rates are all under 0.01. As shown in Figure 3, there are more 95% CIs to the left of the true ${\beta}_{XY}=0.2$ for the MR estimates using external or jackknife resampled PRS across different *P*-value thresholds, and the distributions of intervals are generally balanced in results generated using a stringent *P*-value of 5 × 10^−8^. Conversely, 95% CIs generated using overlapping samples are more to the right of the true ${\beta}_{XY}$ across all *P*-value thresholds, indicating that this method is more likely to overestimate the causal effect of the exposure on the outcome.

For the full summary statistics of this simulated analysis, see Supplementary Material, Tables S1 and S2.

#### Differing sample sizes for external GWAS

Many published trait and disease GWAS do not include UKB, but these typically have smaller sample sizes, which is a key determinant of the power of GWAS and the number of trait-associated SNPs detectable from such studies (13). Thus, to further evaluate the optimal situation for applying the block jackknife resampling MR, we performed another simulation analysis to compare it with classic one-sample MR using PRS constructed with SNPs identified through external GWAS with a small sample size (compared with the applied dataset) as the genetic instruments for the exposure.

In general, the adjusted correlation coefficient $\hat{R}^2$ between PRS and exposure increased with the increase of the sample size of external GWAS in results generated using externally weighted PRS. Using a MR level *P*-value threshold (i.e. *P* < 5 × 10^−8^), block jackknife resampling PRS on an applied dataset of 50 000 individuals explained higher variance in the exposure (mean $\hat{R}^2=0.030$, $bias\big[ MCse\big]=-0.070\big[1.289\times{10}^{-4}\big]$) compared with a weighted score generated using external GWAS with a sample size of 40 000 or less (e.g. when n = 40 000, mean $\hat{R}^2=0.027$, $bias\big[ MCse\big]=-0.073\big[1.229\times{10}^{-4}\big]$). In one-sample MR, median effect estimates ${\beta}_{XY}$ are relatively consistent across results generated with different external GWAS size and the block jackknife resampling framework. For the full simulation metrics, see Supplementary Material, Tables S3 and S4.

#### Exacerbated overfitting with the increase in genetic variants

To evaluate the impact of block number on estimates, we performed the simulation analysis again under 30 different scenarios each using a different number of genetic variants in simulated genotype data (100, 200, 300, 400, 500, 800, 1000, 1200, 1500 and 2000) and a different *P*-value threshold (5 × 10^−8^, 0.05 and 1) for PRS construction, where each analysis was repeated 300 times. Results show that overfitting, measured by the divergence between the estimates generated using overlapping sample PRS and externally weighted PRS, becomes a larger issue with the increase of genetic variants simulated (Supplementary Material, Fig. S1). Overfitting was the most detrimental when constructing PRS using all genetic variants (i.e. with the *P*-value threshold of 1), whereas the results from block jackknife resampled PRS showed a high consistency with the externally weighted scores in all scenarios. In addition, a higher number of SNPs involved in GWAS also resulted in higher variations in the MR estimates derived using the PRS generated under a MR level *P*-value threshold (i.e. *P* < 5 × 10^−8^), regardless of the method used for PRS construction. It is also noteworthy that the coverage of 95% CIs of 2SLS estimates generated with the overlapping sample PRS decreases dramatically with the increase of simulated SNP number, from 0.94 (MCse = 0.014) when N_snp_ = 100 to 0.58 (MCse = 0.028) when N_snp_ = 2000. The decrease in coverage, together with the consistency in bias-eliminated coverage (minimum 0.93) for overlapping sample estimates across all simulated scenarios, suggests that MR using overlapping sample PRS as the IV is severely biased, especially with the increase in SNP number involved in GWAS.

For the full simulation metrics, see Supplementary Material, Tables S5 and S6.

#### The choice of blocks for block jackknife resampling MR

In all analyses (both simulation and applied examples) performed in this study, we applied a fixed number of jackknife blocks (N_block_ = 10) to generate the block jackknife resampled PRS. We conducted simulation to justify this choice as well as to provide insight for readers to appropriately select this parameter for their future analysis. To evaluate the impact of block number on estimates of interest, we performed the simulation analysis again under 21 different scenarios each using a different number of blocks (3, 6, 10, 20, 30, 50 and 100) and a different *P*-value threshold (5 × 10^−8^, 0.05 and 1) for PRS construction, with each analysis being repeated 100 times. Results show that the correlation coefficient ${\hat{R}}^2$ between PRS generated with a different block number and the exposure phenotype and the model-based standard error of causal effect estimates are non-linearly correlated with the block number (Supplementary Material, Fig. S2). As the number of blocks increased from 3 to 10, the correlation coefficient ${\hat{R}}^2$ and model-based standard error generated using the block jackknife resampled PRS changed to become similar to the estimates generated using external PRS, whereas the performance did not change dramatically when using 10 or more blocks to derive jackknife resampled scores. The relationship between block number and estimates was consistent across the three different *P*-value thresholds used for PRS construction. In addition, we observed no clear difference in MR effect estimates (${\hat{\beta}}_{XY}$) and the coverage from results generated using different numbers of blocks. For the full simulation metrics, see Supplementary Material, Tables S7 and S8.

### Applied examples

#### Applied example 1: effect of BMI on circulating biomarkers

**Figure 4:**
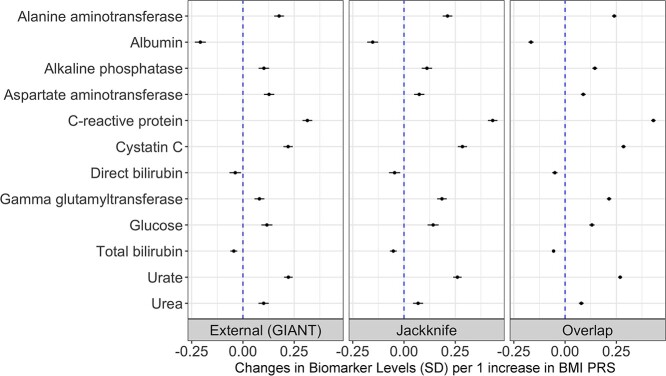
Univariable one-sample MR estimates from BMI and 12 serum biomarkers. One-sample MR estimates and their 95% CIs of effects from the genetic liability towards a high BMI on the levels of 12 biomarkers in the UK Biobank study. External (GIANT) refers to the results generated using externally weighted BMI PRS constructed with Locke *et al.* GWAS summary statistics. Jackknife refers to the results generated using block jackknife resampling BMI PRS constructed with UKB data. Overlap refers to the results generated using internally weighted BMI PRS constructed from a GWAS of overlapping samples for UKB participants.

One-sample MR using three sets of BMI PRS provided generally consistent evidence in the effect from BMI on circulating biomarkers of interest, as shown in forest plots in Figure 4.

BMI provided evidence of a genetically predicted effect on all 12 biomarkers (based on false discovery rates [FDR] < 5%), with C-reactive protein (CRP) having the strongest evidence of an effect in all scenarios (externally weighted PRS: beta = 0.31 SD change in the levels of CRP per 1 increase in BMI PRS, 95% CI = 0.29 to 0.34, *P* = 4.57 × 10^−147^; block jackknife resampled PRS: beta = 0.43, 95% CI = 0.41 to 0.46, *P* = 1.96 × 10^−288^; sample overlapping PRS: beta = 0.43, 95% CI = 0.42 to 0.44, *P* < 1 × 10^−300^). The effect estimates for BMI on each of the 12 biomarkers were generally consistent between three PRSs (Supplementary Material, Table S9). In contrast, effect estimates for BMI instrumented using overlapping the sample score typically had much smaller standard errors (average $\hat{SE}$ =5.83 × 10^−3^), resulting in narrower CIs compared with the other two sets of PRS (externally weighted PRS: average $\hat{SE}$ = 1.24 × 10^−2^; block jackknife resampled PRS: average $\hat{SE}$ =1.22 × 10^−2^).

PRS generated by the three methods all provided strong instruments for BMI. Their *F*-statistics ranged between 4707 and 5695 when using PRS constructed by the Locke *et al.* GWAS, between 5000 and 5857 when using block jackknife resampled PRS and between 23 339 and 27 336 when using PRS generated by GWAS on overlapping samples.

#### Applied example 2: Effect of childhood adiposity on adult testosterone levels

**Figure 5:**
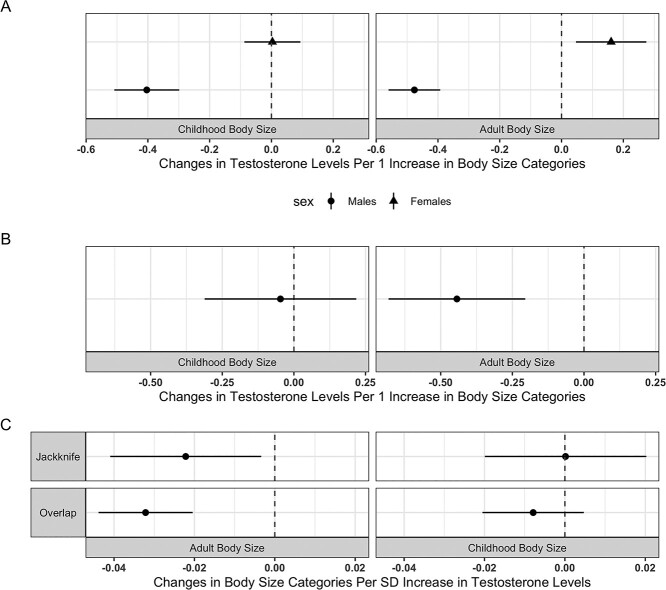
Univariable and multivariable MR on the direct and indirect effects from childhood body size on circulating testosterone levels. (**A**) Sex-stratified univariable one-sample MR estimates between childhood and adult body sizes on circulating testosterone levels in males and females. (**B**) Multivariable one-sample MR estimates between childhood and adult body sizes on circulating testosterone levels in males. (**C**) One-sample MR estimates between the levels of testosterone on childhood and adult body sizes in males, using block jackknife resampling PRS (‘Jackknife’) and sample overlapping PRS (‘Overlap’) for the levels of testosterone as genetic instruments. SD, standard deviation.

Univariable one-sample MR provided strong evidence of a genetically predicted effect between a childhood body size and the levels of testosterone in males (beta = −0.40 SD change in the levels of testosterone per 1 increase in body size category, 95% CI = −0.51 to −0.29, *P* = 4.56 × 10^−14^), whereas there was little evidence of an effect in females (beta = 3.19 × 10^−3^, 95% CI = −0.09 to 0.09, *P* = 0.945) (Fig. 5A). We also found evidence of an effect of genetically predicted adult body size on the levels of testosterone in both sexes based on univariate MR. Higher adult body size had a genetically predicted effect on lower testosterone levels in males (beta = −0.48, 95% CI = −0.56 to –0.39, *P* = 4.01 × 10^−29^), whereas the opposite direction of effect on testosterone was found in females (beta = 0.016, 95% CI = 0.05 to 0.27, *P* = 0.006) (Fig. 5A).

The testosterone lowering effect of higher genetically predicted childhood body size observed in males in the univariable analysis was further explored in a multivariable setting. Results from multivariable MR did not support a direct effect of childhood body size on adulthood testosterone levels after accounting for the genetically predicted effect of adult body size in males (beta = −0.05 per 1 increase in childhood body size category when accounting for adult body size, 95% CI = −0.31 to 0.22, *P* = 0.728). However, there was evidence for an indirect negative effect putatively mediated along the causal pathway involving adult body size (beta = −0.44 per 1 increase in adult body size category when accounting for childhood body size, 95% CI = −0.68 to −0.20, *P* = 2.66 × 10^−4^) (Fig. 5B).

The relationship between body size and the levels of testosterone in males was further assessed using MR in the reverse direction. Results from MR using block jackknife resampling PRS as the genetic instrumental variable provided little evidence for an effect from genetically predicted higher levels of testosterone on childhood body size (beta = 1.62 × 10^−4^ per 1 SD increase in the levels of testosterone, 95% CI = –0.02 to 0.02, *P* = 0.987), while they supported a marginal effect on adult body size (beta = −0.02, 95% CI = –0.04 to −3.38 × 10^−3^, *P* = 0.021). Despite the slight differences in effect estimates, MR using PRS constructed using GWAS on overlapping samples provided results consistent with those from our method (Supplementary Material, Table S10C) (Fig. 5C).

Block jackknife resampling PRS provided strong instruments for childhood and adult body size as well as the levels of testosterone. In females, the F-statistics for childhood and adult body size in the univariable setting are 1003 and 581, respectively. In males, the conditioned F-statistics (in multivariable MR) are 629 and 872 for childhood and adult body size, respectively. In reverse MR analysis, the F-statistics for the levels of testosterone in males is 4342. PRS generated using GWAS on overlapping samples provided a F-statistics of 11 313 for the levels of testosterone in males. Detailed results from the applied example 2 are in Supplementary Material, Table S10.

## Discussion

Sample overlap between multiple GWAS studies is becoming increasingly prevalent owing to the growth of GWAS meta-analyses in recent years. When estimating the causal effect of exposures on continuous outcomes using MR approaches such as 2SLS or the inverse-variance weighted methods, bias owing to sample overlap was shown to be linearly correlated with the percentage of overlap between samples in previous literature (6). However, this bias was not found for binary outcomes when the first-stage regression of 2SLS is conducted using risk factor data on individuals in the control group (6). Various methods have been proposed to account for overlapping participants in summary-data-based MR (14–16), whereas there are limited solution using individual-level data. In this study, we examined biases owing to participant overlap in individual-level one-sample MR results on a continuous outcome and evaluated the effectiveness of a block jackknife resampling MR method in mitigating this bias with both simulated datasets and real data from the UKB. By applying block jackknife resampling MR to investigate the causal effect of genetically predicted childhood body size on adult serum testosterone levels, we demonstrated the application of this approach in terms of enabling sex-stratified, multivariable and bidirectional MR analyses in the absence of an external dataset.

Having an elevated BMI has been linked to multiple health conditions and disease endpoints in multiple observational and MR studies (17–22). Using the block jackknife resampling MR approach outlined in this manuscript, we were able to replicate previous identified effects of higher BMI on the levels of various serum biomarkers which are routinely measured in a clinical setting, including CRP (23), cystatin C (24), alanine aminotransferase and gamma glutamyltransferase (25) as well as urate (26). Together with results from simulation analyses, this first applied example on BMI and biomarkers validated the robustness of block jackknife resampling MR in providing equivalent causal inference compared with classic MR using individual level data and externally identified genetic instruments. In this applied analysis we did not find evidence of inflated 2SLS estimates using the overlapping sample PRS, although our simulation analyses suggest that this may become more problematic when a larger number of SNPs are leveraged as genetic instruments in an MR framework. However, both simulation and applied analyses in UKB show that the main difference between 2SLS estimates generated from overlapping sample PRS and the jackknife PRS is the standard error of effect estimates. The biased 2SLS regression using overlapping sample PRS as the IV produces smaller standard errors, which will lead to narrower confidence intervals and therefore give overprecise (and potentially false-positive) estimates. Our findings from simulated data also suggest that the block jackknife resampling study design is preferable in situations where (1) you have a large dataset containing both exposure and outcome data that can be used to derive genetic instrument variables and perform MR and (2) the only available external GWAS is substantially smaller. Moreover, using 10 blocks for jackknife resampling is recommended based on the trade-off between the integrity of results and computational cost.

Despite the increase in GWAS meta-analysis for common traits and diseases such as BMI and type 2 diabetes, numerous phenotypes are underinvestigated in GWAS and MR owing to limited data available in non-overlapping samples. In the second applied example undertaken by our study, we examined the sex-specific causal effect from childhood adiposity on adult serum testosterone levels, as an illustration of how block jackknife resampling MR could be applied to study causal relationships involving phenotypes with limited data sources. Testosterone is a sex hormone produced predominately in males and plays important roles in the development of masculine characteristics. Low levels of testosterone produced in the body, namely testosterone deficiency or hypogonadism, is a condition that primarily affects older men (27). This condition is often treated with testosterone replacement therapy; however, concerns regarding the safety of this therapy have been raised as adverse events following its use have been reported (28–30). Therefore, identifying any modifiable risk factors contributing to changes in testosterone levels would be valuable for preventing this condition. Childhood-onset adiposity, previously identified as an early life risk factor for multiple cardiovascular diseases and cancer (31,32), was reported to associate with lower testosterone levels in adulthood in an observational study (33). This association could be owing to confounding and thus should be further studied using MR to determine whether childhood adiposity has a causal effect on lower levels of testosterone.

Independent SNPs that reached genome-wide significance (*P* < 5 × 10^−8^) in GWAS are often selected as candidate IVs for MR. GWAS with a larger sample size usually have more power to identify SNPs associated with traits or diseases, providing stronger instruments for MR. Meanwhile, a dataset for one-sample MR analysis also needs to be large enough to provide statistical power for identifying any causal relationship between the exposure and the outcome. To date, the largest datasets on childhood adiposity (proxied by recalled body size compared with others at 10 years old) and serum levels of testosterone were both available in UKB. With the advantage of a large sample size, GWAS on UKB traits can provide higher statistical power for subsequent MR analysis by enabling use of a larger number of instruments compared with those available externally, but they have the issue of participant overlap between the samples for IV discovery and MR. Instead, using block jackknife resampling MR, we were able to identify sex-specific genetic IVs for childhood body size and examine the sex-stratified causal effect from childhood adiposity on adult serum testosterone using the largest dataset available without violating the independence assumption of MR or losing much power. Univariable and multivariable MR support an indirect causal effect of higher childhood body size on lower testosterone in men mediated by higher adult body size, consistent with a previous finding that higher adult BMI is causally associated with lower testosterone levels in men (34). This suggests that the influence of childhood obesity on lower serum testosterone could be mitigated if one loses weight in adulthood, similar to previously identified causal effects of early life and adult body size on type 2 diabetes and risk of coronary artery disease (35). This applied example illustrated the value of this block jackknife resampling study design in a causal inference setting when using limited sources of data for sex-stratified IV discovery, as well as when applying MR in multivariable and bidirectional settings.

This method has some limitations. First, the identification of block jackknife resampled genetic instruments requires a higher computational burden than in classic MR where IVs were identified in one GWAS study, although the proposed method is expected to be less computational expensive than the Bayesian approach accounting for participant overlap (15). Second, the block jackknife resampling framework requires access to individual level phenotype and genotype data to enable the construction of allele scores for every participant in the sample. Despite this, our findings show that the implementation of block jackknife resampling MR successfully addresses any biases arise from 100% sample overlap between the IV discovery dataset and the applied dataset. This is advantageous over the existing summary-data based MR method MRlap, which outperforms all classic MR methods when the overlap is 100%, whereas the MR estimates are still inflated when compared with results generated under the non-overlapping two-sample settings (14). Third, through block jackknife resampling MR we cannot directly identify the existence of horizontal pleiotropic SNPs. Horizontal pleiotropy arises when one or more SNPs used as the genetic instrumental variable for the exposure influence the outcome through a pathway which does not involve the exposure. The inclusion of such SNPs as genetic instrumental variables for an exposure can reintroduce confounding and lead to bias in causal inference (5). Horizontal pleiotropy could be detected through detailed examination of individual SNPs used for PRS construction in each block separately, through evaluating the between-SNP heterogeneity in the ratio of associations between genotype and the outcome and the exposure (36,37).

In summary, block jackknife resampling MR method provides researchers with an approach to perform hypothesis testing with limited sources of data before conducting a comprehensive assessment of causal relationships between modifiable risk factors and complex disease traits and outcomes.

## Materials and Methods

### Description of the block jackknife resampling MR method

Block jackknife resampling MR is a modified approach to one-sample MR which uses individual-level genotype and phenotype data to study the causal relationship between risk factors (exposures) and diseases or traits (outcomes) in a single dataset. It provides maximum statistical power for causal inference while avoiding biases owing to participant overlap between the datasets for IV discovery and for causal inference.

Performing a block jackknife resampling MR study involves the following steps:

1. Split the full dataset randomly into N_block_ groups.
2. Perform N_block_ GWAS on the exposure of interest using all samples from each permutation set of N_block_-1 groups to obtain genome-wide corrected SNPs (i.e. *P* < 5 × 10^−8^) and weights for PRS construction across each set.
3. Construct PRS for the exposure for individuals with each of the N_block_ groups based on the SNPs and weights identified in the GWAS which they were not analyzed as part of (similar to a cross-validation approach).
4. Combine all groups together and use the PRS as a genetic IV for the exposure in one-sample MR using 2SLS regressions.

With the availability of additional phenotype data, the block jackknife resampling framework can be extended to evaluate direct and indirect effects for the exposure of interest in a multivariate setting. This approach can also be applied to investigate reverse causation when GWAS and PRS constructed are performed on the outcome being assessed.

### Simulation analyses

To investigate the effect of sample overlap and the advantages of using a block jackknife resampling framework to mitigate overfitting bias, we performed extensive simulations to construct PRS under the combination of three GWAS frameworks and 13 different *P*-value thresholds. Figure 1 shows a schematic illustration of the three approaches.

We generated 1000 pairs of simulated datasets consisting of n = 50 000 (referred to as our ‘applied dataset’) and n = 50 000 (referred to as our ‘external dataset’) individuals. In the applied datasets, three continuous phenotypes (i.e. exposure X_1_, outcome Y and confounder U_1_) and genetic data consisting of 500 independent SNPs were simulated for every individual. In the external datasets, two continuous phenotypes (i.e. exposure X_2_ and confounder U_1_) and genetic data consisting of 500 SNPs were simulated for every individual. Data simulation was achieved using the ‘stats’ and ‘simulateGP’ R package (https://github.com/explodecomputer/simulateGP/) Supplementary Material, Methods and Supplementary Material, Figure S3. A set of fixed parameters were applied, including the total variance in the exposure explained by all SNPs (Var_exp_ = 0.1), effect allele frequency (AF) of all SNPs (AF = 0.2), the effect from the exposure to the outcome (${\beta}_{XY}=0.2$), the effect from the confounder to the exposure (${\beta}_{UX}=0.4$) and the effect from the confounder to the outcome (${\beta}_{UY}=0.3$).

Using the simulated data described before, GWAS of the exposure variables were conducted (A) using the external dataset (referred to as ‘external GWAS’), (B) using all samples in the applied dataset (referred to as ‘overfitted GWAS’) and (C) following a block jackknife resampling framework in the applied dataset (referred to as ‘jackknife GWAS’) (Fig. 1). GWAS on simulated data were performed using linear regression as implemented through the ‘gwas’ function in the ‘simulateGP’ R package.

After generating GWAS summary statistics of the exposure, SNPs were filtered based on their genome wide significance level (i.e. *P*-value) using one of the 13 different *P*-value thresholds (5 × 10^−8^_,_ 1 × 10^−7^_,_ 1 × 10^−6^, 1 × 10^−5^, 1 × 10^−4^, 5 × 10^−4^, 1 × 10^−3^, 5 × 10^−3^, 0.01, 0.05, 0.1, 0.5 or 1). For individuals in the applied datasets, three PRSs were constructed as the weighted sum of the number of effect alleles of SNPs that reached the *P*-value threshold. LD clumping was not undertaken on simulated GWAS statistics because all SNPs were simulated to be uncorrelated.

The adjusted correlation coefficient $\hat{R}^2$ between PRS and the exposure was calculated using linear regression in the applied dataset for each of the three simulated PRS in turn. Estimates (beta coefficients $\hat{\beta}_{XY}$, and their standard errors $\hat{SE}\big(\hat{\beta}_{XY}\big)$) of the genetically predicted effect of exposures on the simulated outcomes were calculated via 2SLS regression. This was achieved using the ivreg() function from the ‘ivpack’ R package. The R package ‘rsimsum’ (38) was used to compute simulation metrics including the mean, median, bias, empirical standard error, percentage gain in precision relative to the externally weighted PRS, mean squared error for adjusted $\hat{R}^2$ and $\hat{\beta}_{XY}$, the mean, median and relative percentage error in standard error for $\hat{\beta}_{XY}$, the model-based standard error and relative error in model-based standard error for $\hat{\beta}_{XY}$, the coverage and bias-eliminated coverage for $\hat{\beta}_{XY}$, and Monte Carlo standard error of all summary statistics where applicable. Details of these statistical measures have been described previously (39).

To further evaluate the optimal situation for applying the block jackknife resampling MR, we performed another simulation analysis to compare it with classic one-sample MR using PRS constructed with SNPs identified through external GWAS with a small sample size (compared with the applied dataset) as the genetic instruments for the exposure.

Using the same approach used in the first simulated analysis, we generated phenotype and genotype data for 1000 pairs of applied datasets (n = 50 000) and external datasets with each of the different sample sizes (n = 10 000, 15 000, 20 000, 25 000, 30 000, 35 000, 40 000, 45 000 and 50 000). GWAS and PRS construction were undertaken using the three frameworks performed in the first analysis, except all PRS were calculated using a stringent *P*-value threshold for MR (*P* < 5 × 10^−8^). Linear regression and 2SLS were performed to generate the correlation coefficients and estimate of the effects from the exposure to the outcome. The R package ‘rsimsum’ (38) was used to compute simulation metrics mentioned in the first analysis.

To evaluate whether the number of SNPs simulated affect the results, we generated another set of phenotype and genotype data for 300 pairs of applied datasets (n = 50 000) and external datasets (n = 50 000) with varying numbers of SNPs (N_snp_ = 100, 200, 300, 400, 500, 800, 1000, 1200, 1500 and 2000) together explaining 10% of the variances in the exposure (i.e. true ${R}^2=0.1$). GWAS, PRS construction and association analysis were performed using the same methods mentioned before. The resulting correlation coefficients and effect estimates were examined using simulation metrics as described previously.

In addition, we applied the number of 10 blocks across all analyses in this study. To investigate whether the choice of block number would lead to differences in estimates, we performed additional simulation using the same parameters as the primary analysis and calculated the block jackknife resampled PRS using seven different number of blocks (N_block_ = 3, 6, 10, 20, 50, 100) and three different *P*-value threshold (5 × 10^−8^, 0.05 and 1). The resulting correlation coefficients and effect estimates were compared with those generated for the sample overlapping PRS and externally weighted PRS, and they were evaluated using the simulation metrics as described previously.

### Applied examples

#### Effect of BMI on circulating biomarkers

In the first applied analysis, we implemented the block jackknife resampling MR together with the two other approaches used in the simulated analysis to examine the causal effects from body mass index on a set of 12 circulating biomarkers using data from the UKB.

UKB is a large-scale prospective cohort study consisting of approximately 500 000 individuals aged between 38 and 73 years at baseline from across the United Kingdom (40). Data were collected based on clinical examinations, assays of biological samples, questionnaires and interviews, as well as genome-wide genotyping as described previously (41,42). UKB received ethical approval from the Research Ethics Committee (REC reference 11/NW/0382).

BMI was calculated by weight (kg) divided by standing height (m) squared, both measured at the initial assessment. We focused on a subset of circulating biomarkers which were all measured from serum samples also obtained at baseline (C-reactive protein, alkaline phosphate, testosterone, glucose, cystatin C, urea, urate, albumin, direct bilirubin, total bilirubin, gamma glutamyltransferase, alanine aminotransferase, aspartate aminotransferase).

In this applied analysis, we constructed a BMI PRS using data from UKB participants (n = 333 894) with BMI associated genetic variants identified and their weights estimated using three GWAS frameworks (Fig. 1):

1. Using summary statistics of the Locke *et al.* BMI GWAS meta-analysis (43) which has no reported sample overlap with the UKB to generate an externally weighted PRS;
2. Using the full UKB cohort to perform BMI GWAS and generate sample overlapping PRS;
3. Using the full UKB cohort to perform BMI GWAS with the block jackknife resampling framework and generating a block jackknife resampled PRS.

In scenario 1, the weighted PRS for BMI was constructed using genome-wide significant (*P* < 5 × 10^−8^) genetic variants from the summary statistics of the Locke *et al.* BMI GWAS meta-analysis on up to 322 154 individuals of European descent. In scenario 2, a GWAS for BMI was conducted on 461 377 UKB participants using a linear mixed model (LMM) association method as implemented in BOLT-LMM (v2.3) (44) to account for population structure in the UKB. Age, sex and genotyping chip were included as covariates. The GWAS results were clumped using a reference panel consisting of a subset of unrelated UKB participants of European ancestry (N = 10 000), as described in a previous study (45) to identify independent genetic variants (linkage disequilibrium threshold *r*^2^ < 0.001 within a 1000 kb region) which reached the genome-wide significance (*P* < 5 × 10^−8^). Those independent variants were used to construct weighted PRS for BMI. Clumping and PRS construction were achieved using PLINK (v2.0) (46). In scenario 3, we first randomly assigned the 461 377 UKB participants with BMI phenotype into 10 groups. GWAS was undertaken on each possible set of nine groups with adjustment for the same covariates as in scenario 2. GWAS results were clumped and then used to generate weighted PRS for individuals in the remaining group for each set. Details of the GWAS pipeline can be found in Supplementary Methods.

Using a BMI PRS generated through three frameworks as described before, we performed one-sample MR to examine the relationship between BMI and 12 circulating biomarkers (CRP, alkaline phosphate, glucose, cystatin C, urea, urate, albumin, direct bilirubin, total bilirubin, gamma glutamyltransferase, alanine aminotransferase, aspartate aminotransferase) in the UKB. Biomarker levels underwent rank-based inverse normal transformation before MR analysis to ensure their normality. We applied 2SLS regressions using the BMI PRS as a genetic instrument, with sex, age and the first 10 principal components fitted as covariates. This allowed us to compare estimates from the three different PRS generated using each of the scenarios described before.

#### Effect of childhood adiposity on adult testosterone levels

We applied block jackknife resampling MR to investigate sex-specific effects of childhood adiposity on serum testosterone levels measured in adults in the UKB through (1) univariable MR, (2) multivariable MR and (3) bidirectional MR, all in a one-sample setting.

Childhood body size was derived using questionnaire data asking participants to recall their body size at 10 years old as ‘thinner’ or ‘plumper’ than average, or ‘about average’, as described previously (35). The robustness of using allele scores constructed with SNPs associated with this childhood body size phenotype has been validated in three independent cohorts previously (35,47,48). For comparative purposes, adult body size was derived by categorizing the BMI data into a three-category variable using the same proportions as seen in the strata of the childhood body size variable. Before analysis, the UKB measurement of circulating testosterone was stratified by sex and then transformed using a rank-based inverse normal transformation.

Block jackknife resampling was conducted in female-only and male-only UKB participants separately to construct sex-specific PRS for childhood and adult body size as well as the levels of circulating testosterone. UKB samples were randomly assigned into 10 groups for both males and females. GWAS on the three phenotypes were undertaken on participants from each of the nine groups adjusted for age and genotyping chip, and the results were used to construct PRS for individuals in the remaining group.

Next, we performed univariable one-sample MR using sex-specific PRS for childhood body size as genetic instruments. 2SLS regressions were undertaken for males and females separately, where age and the first 10 principal components were fitted as covariates. To assist with comparisons between adult and childhood body size, we also estimated the effects from the comparable three-tier adult body size variable on testosterone levels using the univariable model.

Moreover, we used multivariable one-sample MR to estimate the direct and indirect role of childhood body size on testosterone levels where evidence of a total effect was identified in univariable analyses. This was achieved by accounting for adult body size as an additional exposure in the 2SLS analysis.

Finally, we performed one-sample MR in the reverse direction, i.e. using the levels of testosterone as the exposure and childhood/adult body size as the outcome, to examine whether genetically predicted levels of testosterone in adulthood influences either childhood body size (i.e. as a negative control) or adult body size. One-sample MR using 2SLS was performed, where age and the first 10 principal components were fitted as covariates. Results were obtained from MR using block jackknife resampling PRS and using overlapping sample PRS (constructed using SNPs and weights from a GWAS on all UKB participants) as the genetic instruments for the levels of circulating testosterone.

All statistical analyses were undertaken using R (v4.0.2) (49).

## Supplementary Material

Fang_JackknifeResamplingMR_SupplementaryInformation_R2_ddac186

Fang_JackknifeResamplingMR_SupplementaryInformation_R2_withTrackedChanges_ddac186

Fang_JackknifeResamplingMR_SupplementaryTables_R1_ddac186
