# Supplementary material for: Evaluating and implementing block jackknife resampling Mendelian randomization to mitigate bias induced by overlapping samples
Source: Hum Mol Genet. 2022 Aug 6;32(2):192–203. doi: 10.1093/hmg/ddac186 (PMC9840213; doi:10.1093/hmg/ddac186)
Supplement: Fang_JackknifeResamplingMR_SupplementaryInformation_R2_ddac186 [file fang_jackkniferesamplingmr_supplementaryinformation_r2_ddac186.zip › Fang_JackknifeResamplingMR_SupplementaryInformation_R2_ddac186.docx]

**Evaluating and implementing block jackknife resampling Mendelian randomization to mitigate bias induced by overlapping samples**

Supporting Information

Table of Contents

[Supplementary Methods 3](#_Toc103947495)

[Simulation analysis 3](#_Toc103947496)

[Two-stage least squares regression 4](#_Toc103947497)

[The UK Biobank study 5](#_Toc103947498)

[Genotyping and imputation 5](#_Toc103947499)

[Data quality control 6](#_Toc103947500)

[Association analysis: statistical methods 6](#_Toc103947501)

[Supplementary Figures 7](#_Toc103947502)

[Figure S1. Results from simulation analyses investigating the impact of the number of SNPs involved in GWAS for genetic instrument discovery on results. 7](#_Toc103947503)

[Figure S2. Results from simulation analyses investigating the impact of the number of jackknife blocks involved in GWAS for genetic instrument discovery on results. 9](#_Toc103947504)

[Figure S3. A directed acyclic graph showing the associations between variables involved in data simulation. 11](#_Toc103947505)

[Supplementary Tables 12](#_Toc103947506)

[Table S1. Summary statistics for the correlation coefficient (R^2^) between the simulated exposure and PRS in the primary simulation analysis comparing three methods for GWAS and PRS construction. 12](#_Toc103947507)

[Table S2. Summary statistics for the two-stage least squares effect estimate (beta coefficient) of the exposure on the outcome in the primary simulation analysis comparing three methods for GWAS and PRS construction. 12](#_Toc103947508)

[Table S3. Summary statistics for the correlation coefficient (R^2^) between the simulated exposure and PRS in the second simulation analysis comparing block jackknife resampled PRS, overlapping sample PRS and externally weighted PRS generated using external GWAS in small sample sizes. 13](#_Toc103947509)

[Table S4. Summary statistics for the two-stage least squares effect estimate (beta coefficient) of the exposure on the outcome in the second simulation analysis comparing block jackknife resampled PRS, overlapping sample PRS and externally weighted PRS generated using external GWAS in small sample sizes. 13](#_Toc103947510)

[Table S5. Summary statistics for the correlation coefficient (R^2^) between the simulated exposure and PRS in the simulation analysis investigating the impact of the number of SNPs involved in GWAS for genetic instrument discovery on results. 14](#_Toc103947511)

[Table S6. Summary statistics for the two-stage least squares effect estimate (beta coefficient) of the exposure on the outcome in the simulation analysis investigating the impact of the number of SNPs involved in GWAS for genetic instrument discovery on results. 15](#_Toc103947512)

[Table S7. Summary statistics for the correlation coefficient (R^2^) between the simulated exposure and PRS in the simulation analysis investigating the impact of the number of jackknife blocks involved in GWAS for genetic instrument discovery on results. 15](#_Toc103947513)

[Table S8. Summary statistics for the two-stage least squares effect estimate (beta coefficient) of the exposure on the outcome in the simulation analysis investigating the impact of the number of jackknife blocks involved in GWAS for genetic instrument discovery on results. 16](#_Toc103947514)

[Table S9. Results of one-sample MR on the effect of body mass index (BMI) on circulating blood biomarkers in the UK Biobank, where BMI were instrumented by weighted PRS constructed by three different GWAS frameworks. 17](#_Toc103947515)

[Table S10. Results from block jackknife resampling MR on the effects of childhood and adult body size on the levels of testosterone in adulthood. 17](#_Toc103947516)

[References 18](#_Toc103947517)

# Supplementary Methods

## Simulation analysis

Data simulation was conducted using the *stats* and *simulateGP* R package (<https://github.com/explodecomputer/simulateGP>) in R. A set of fixed parameters were applied, including the total variance in the exposure explained by all SNPs (Var_exp_=0.1), effect allele frequency (AF) of all SNPs (AF=0.2), the effect from the exposure to the outcome ($\beta_{XY}=0.2$), the effect from the confounder to the exposure ($\beta_{UX}=0.4$) and the effect from the confounder to the outcome ($\beta_{UY}=0.3$).

As describe in the documentation of the *simulateGP* package (<https://explodecomputer.github.io/simulateGP/articles/simplemr.html>), the simulation of genotype and phenotype data was based on the assumptions of Mendelian randomization, which aims to estimate the causal effect of an exposure X on an outcome Y using a genetic instrumental variable for the exposure G. The assumptions include:

1. G associates with X.
2. G is independent of any confounders of X and Y.
3. G only associate with Y through the effect of X.

In addition, the simulated effect involves influence from confounder U to both X and Y. The relationship between G, X, Y and U is illustrated as a directed acyclic graph (DAG) in **Supplementary Figure S3**. To generate genotype and phenotype data which fulfils these assumptions, data simulation is performed as follows:

1. Simulate the genetic variables and confounding variables.
2. Simulate the exposure which is influence by genotype and confounder from (1).
3. Simulate the outcome which is influence by the confounder from (1) and exposure from (2).

irstly, we simulate the effects from each of the N_snp_ single nucleotide polymorphisms (SNPs) to the exposure phenotype using the choose_effect() function, given the number of SNPs (N_snp_) and total variance explained by all SNPs (Var_exp_). Next, the individual level genotype matrix was generated using the make_geno() function based on sample size (n), N_snp_ as well as a fixed allele frequency of all SNPs. The confounder variable was simulated by generating a set of randomly distributed values with a mean of 0 and standard deviation of 1 using the rnorm() function.

The exposure and outcome variables were both generated using the make_phen() function. The exposure variable was simulated based on the effects from SNPs to the exposure generated in the first step, the effect from the confounder to the exposure ($\beta_{UX}$) and the genotype matrix and the confounder variable. The outcome variable was then simulated based on the exposure variable and the effects from the exposure to the outcome ($\beta_{XY}$), as well as the confounder variable and the effect from the confounder to the outcome ($\beta_{UY}$).

## Two-stage least squares regression

In Mendelian randomization, two-stage least squares (2SLS) is a statistical method commonly used for examining the linear relationship between a continuous exposure X and a continuous outcome Y with the availability of individual-level data (1). 2SLS consists of two linear regressions: (1) the first stage regression generates the fitted values of an exposure by regressing the exposure X on the genetic instrumental variable Z; (2) the second stage regression generates the causal estimates from X to Y by regressing the outcome Y on the fitted values of the exposure from the first stage regression.

In this study, 2SLS regression was performed using the ivreg() function from the *ivpack* R package, which automatically accounts for the uncertainty in the first stage regression.

## The UK Biobank study

The UK Biobank is a population-based health research resource consisting of approximately 500,000 people, aged between 38 years and 73 years, who were recruited between the years 2006 and 2010 from across the UK (2). Particularly focused on identifying determinants of human diseases in middle-aged and older individuals, participants provided a range of information (such as demographics, health status, lifestyle measures, cognitive testing, personality self-report, and physical and mental health measures) via questionnaires and interviews; anthropometric measures, BP readings and samples of blood, urine and saliva were also taken (data available at www.ukbiobank.ac.uk). A full description of the study design, participants and quality control (QC) methods have been described in detail previously (3). UK Biobank received ethical approval from the Research Ethics Committee (REC reference for UK Biobank is 11/NW/0382).

## Genotyping and imputation

The full data release contains the cohort of successfully genotyped samples (n=488,377). 49,979 individuals were genotyped using the UK BiLEVE array and 438,398 using the UK Biobank axiom array. Pre-imputation QC, phasing and imputation are described elsewhere (4). In brief, prior to phasing, multiallelic SNPs or those with MAF ≤1 % were removed. Phasing of genotype data was performed using a modified version of the SHAPEIT2 algorithm (5). Genotype imputation to a reference set combining the UK10K haplotype and HRC reference panels (6) was performed using IMPUTE2 algorithms (7). The analyses presented here were restricted to autosomal variants within the HRC site list using a graded filtering with varying imputation quality for different allele frequency ranges. Therefore, rarer genetic variants are required to have a higher imputation INFO score (Info>0.3 for MAF >3%; Info>0.6 for MAF 1-3%; Info>0.8 for MAF 0.5-1%; Info>0.9 for MAF 0.1-0.5%) with MAF and Info scores having been recalculated on an in-house derived ‘European’ subset (8).

## Data quality control

Individuals with sex-mismatch (derived by comparing genetic sex and reported sex) or individuals with sex-chromosome aneuploidy were excluded from the analysis (n=814). We restricted the sample to individuals of ‘European’ ancestry as defined by an in-house k-means cluster analysis performed using the first 4 principal components provided by UK Biobank in the statistical software environment R. The current analysis includes the largest cluster from this analysis (n=464,708) (8).

## Association analysis: statistical methods

Genome-wide association analysis (GWAS) was conducted using linear mixed model (LMM) association method as implemented in BOLT-LMM (v2.3) (9). To model population structure in the sample, we used 143,006 directly genotyped SNPs, obtained after filtering on MAF > 0.01; genotyping rate > 0.015; Hardy-Weinberg equilibrium p-value < 0.0001 and LD pruning to an r^2^ threshold of 0.1 using PLINKv2.00. Genotype array and sex were adjusted for in the model. BOLT-LMM association statistics are on the linear scale.

# Supplementary Figures


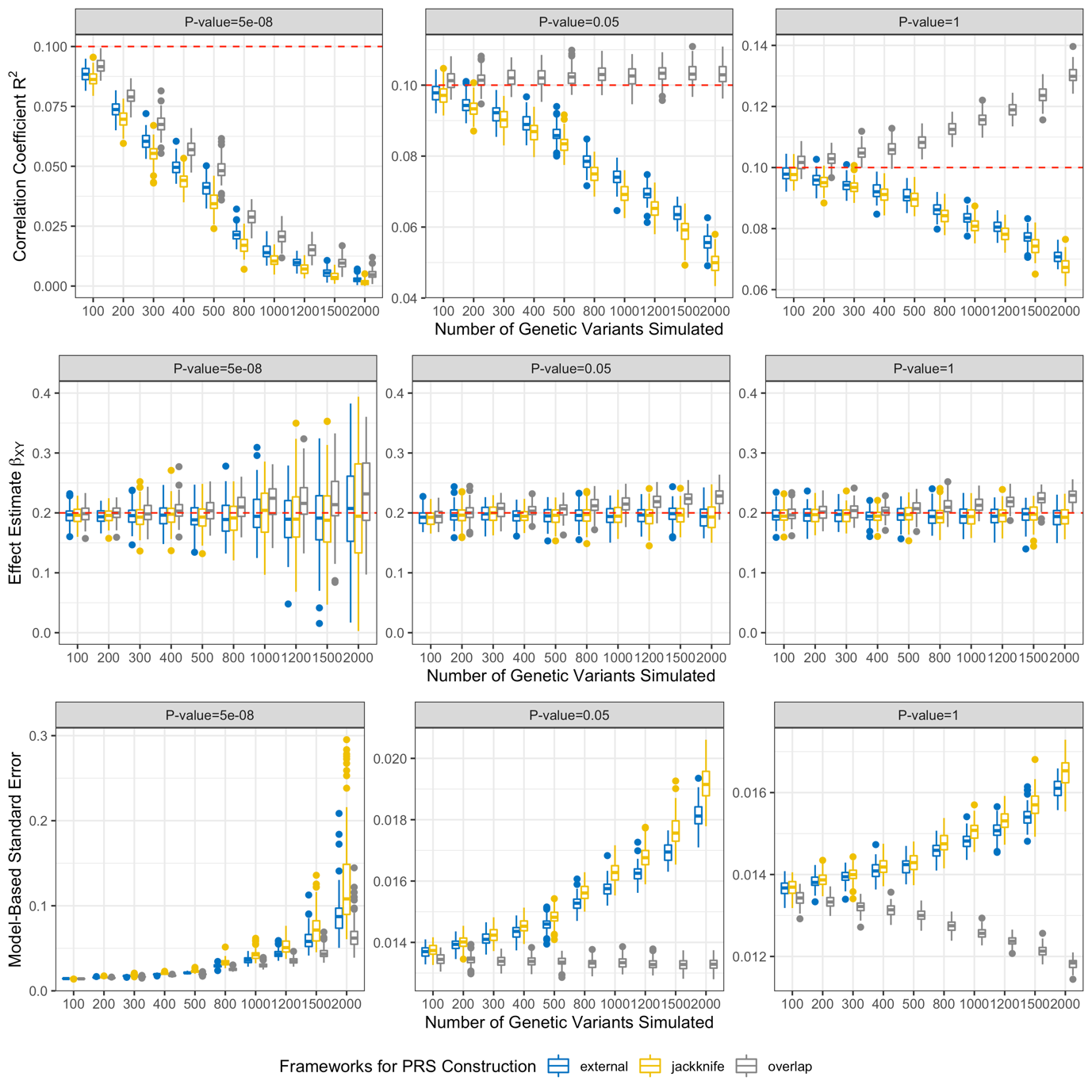


## Figure S1. Results from simulation analyses investigating the impact of the number of SNPs involved in GWAS for genetic instrument discovery on results.

Results from simulations performed using simulated genotypes consisting of different number of SNP ranging from 100 to 2000 can be found on the x-axis. Plots show the performance of each PRS constructed under three GWAS frameworks in terms of phenotype prediction (top) and one-sample Mendelian randomization (middle and bottom). Plots from left to right are results generated using PRS generated with genetic variants clumped by three different P-value thresholds (5x10^-8^, 0.05 and 1 respectively). The plots on the top row are box plots showing the distribution of the correlation coefficients between simulated PRS and exposure. The plots in the middle row are boxplots showing the distribution of MR estimates (beta coefficients) of effect of the exposure on the outcome. The plots on the bottom row are box plots showing the distribution of model-based standard errors of the effect estimates. Red lines represent the parameters used in data simulation, i.e., the true value of correlation coefficient R^2^ (0.10) in plots at the top row and the true effect from the exposure to the outcome $\beta_{XY}$ (0.20) in plots in the middle row. overlap, results generated using overlapping sample PRS. external, results generated using externally weighted PRS. jackknife, results generated using block jackknife resampled PRS.


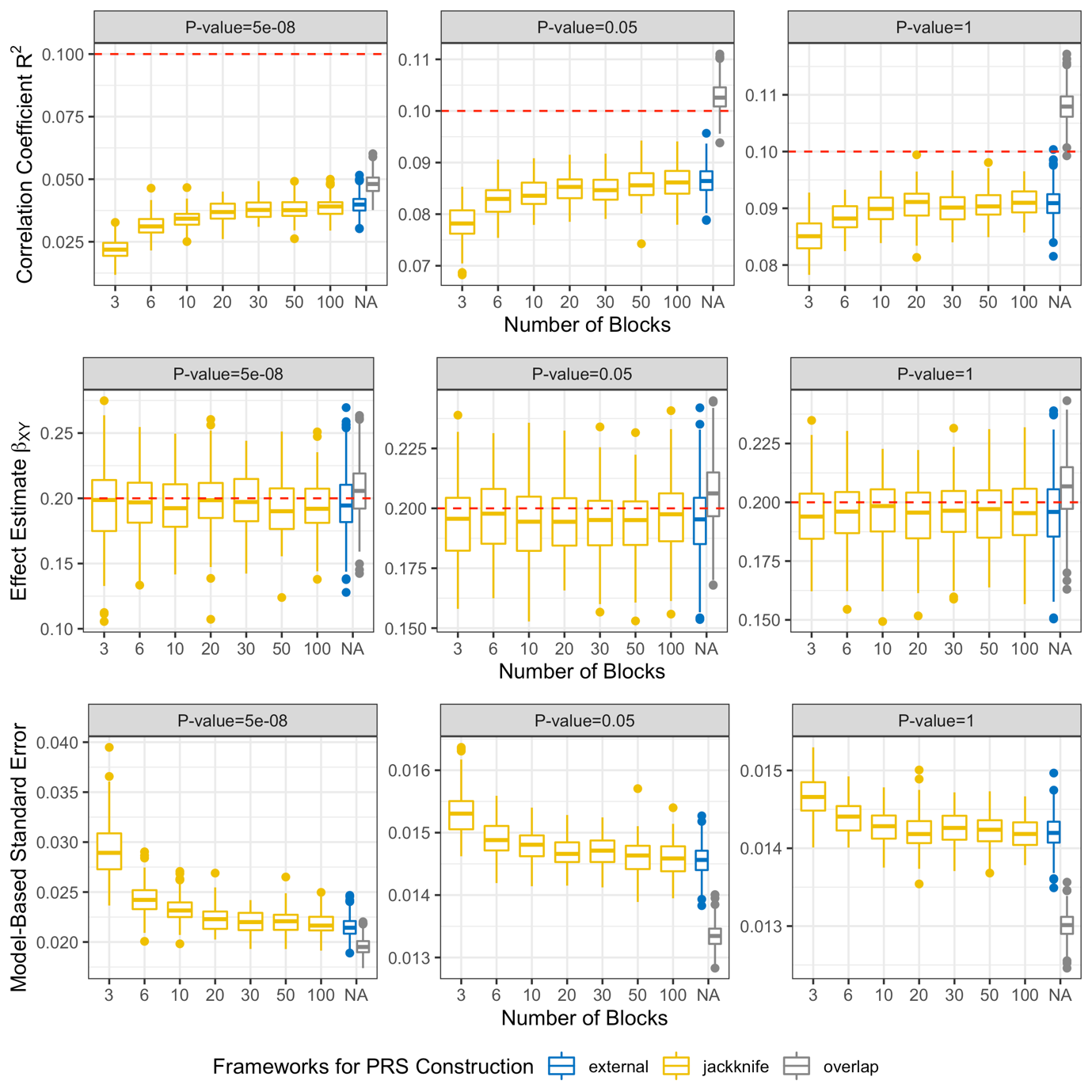


## Figure S2. Results from simulation analyses investigating the impact of the number of jackknife blocks involved in GWAS for genetic instrument discovery on results.

Results in this figure are from simulations performed using PRS constructed using overlapping sample GWAS (“overlap”), external GWAS (“external”), and seven different block jackknife resampled PRSs (“jackknife”) constructed by dividing the full sample into 3 to 100 blocks, as shown in the x-axes. Plots show the performance of each PRS constructed under three GWAS frameworks in terms of phenotype prediction (top) and one-sample Mendelian randomization (middle and bottom). Plots from left to right are results generated using PRS generated with genetic variants clumped by three different P-value thresholds (5x10^-8^, 0.05 and 1 respectively). The plots on the top row are box plots showing the distribution of the correlation coefficients between simulated PRS and exposure. The plots in the middle row are boxplots showing the distribution of MR estimates (beta coefficients) of effect of the exposure on the outcome. The plots on the bottom row are box plots showing the distribution of model-based standard errors of the effect estimates. Red lines represent the parameters used in data simulation, i.e., the true value of correlation coefficient R^2^ (0.10) in plots on the top row and the true effect from the exposure to the outcome $\beta_{XY}$ (0.20) in plots in the middle row.


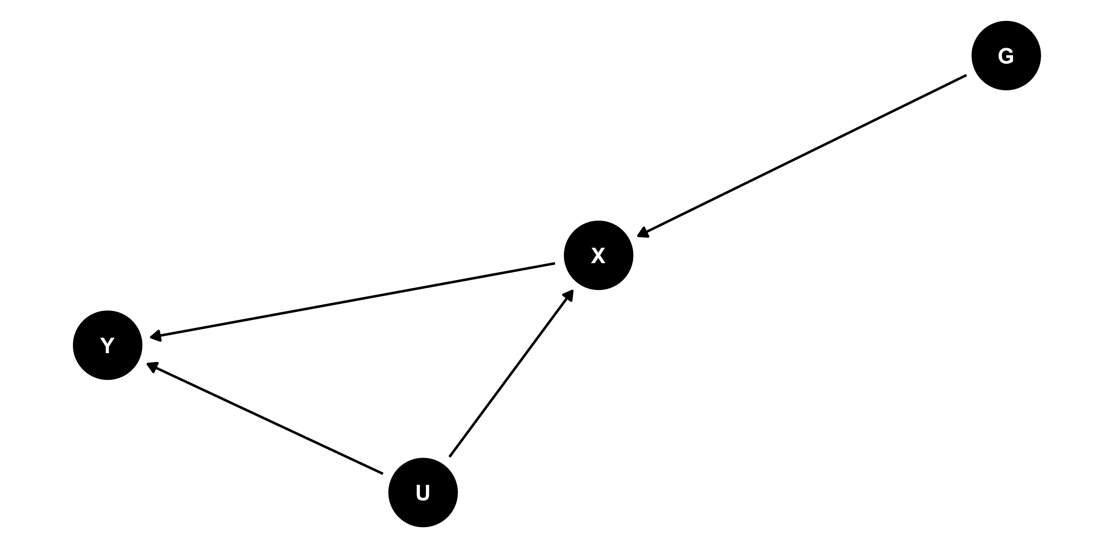


## Figure S3. A directed acyclic graph showing the associations between variables involved in data simulation.

G, genetic instrumental variable. X, the exposure. Y, the outcome. U, a confounding factor which has causal effect on both X and Y.

# Supplementary Tables

## Table S1. Summary statistics for the correlation coefficient (R^2^) between the simulated exposure and PRS in the primary simulation analysis comparing three methods for GWAS and PRS construction.

The correlation coefficient R^2^ is noted as theta in the table. “nsim”, the number of repeated simulation. “thetamean”, mean of correlation coefficient R^2^. “thetamedian”, median of correlation coefficient R^2^. “bias”, bias in correlation coefficient R^2^. “empse”, empirical standard error. “mse”, mean squared error. “relprec”, percentage gain in precision relative to the reference method. “mcse”, Mont Carlo standard errors. “lower” and “upper” denote the lower limit and upper limit of the Mont Carlo 95% confidence intervals respectively.

(See separate excel file)

## Table S2. Summary statistics for the two-stage least squares effect estimate (beta coefficient) of the exposure on the outcome in the primary simulation analysis comparing three methods for GWAS and PRS construction.

The beta coefficient is noted as theta in the table. “nsim”, the number of repeated simulation. “thetamean”, mean of correlation coefficient R^2^. “thetamedian”, median of correlation coefficient R^2^. “se2mean”, average standard error. “se2median”, median standard error. “bias”, bias in correlation coefficient R^2^. “empse”, empirical standard error. “mse”, mean squared error. “relprec”, percentage gain in precision relative to the reference method. “modelse”, model-based standard error. “relerror”, relative percentage error in standard error. “cover”, coverage of a nominal level. “becover”, bias-eliminated coverage of a nominal level. “power”, power of a (1 - level) (defaults to NULL, in which case all summary statistics are returned). “mcse”, Mont Carlo standard errors. “lower” and “upper” denote the lower limit and upper limit of the Mont Carlo 95% confidence intervals.

(See separate excel file)

## Table S3. Summary statistics for the correlation coefficient (R^2^) between the simulated exposure and PRS in the second simulation analysis comparing block jackknife resampled PRS, overlapping sample PRS and externally weighted PRS generated using external GWAS in small sample sizes.

The correlation coefficient R^2^ is noted as theta in the table. “nsim”, the number of repeated simulations. “thetamean”, mean of correlation coefficient R^2^. “thetamedian”, median of correlation coefficient R^2^. “bias”, bias in correlation coefficient R^2^. “empse”, empirical standard error. “mse”, mean squared error. “relprec”, percentage gain in precision relative to the reference method. “mcse”, Mont Carlo standard errors. “lower” and “upper” denote the lower limit and upper limit of the Mont Carlo 95% confidence intervals respectively.

(See separate excel file)

## Table S4. Summary statistics for the two-stage least squares effect estimate (beta coefficient) of the exposure on the outcome in the second simulation analysis comparing block jackknife resampled PRS, overlapping sample PRS and externally weighted PRS generated using external GWAS in small sample sizes.

The beta coefficient is noted as theta in the table. “nsim”, the number of repeated simulation. “thetamean”, mean of correlation coefficient R^2^. “thetamedian”, median of correlation coefficient R^2^. “se2mean”, average standard error. “se2median”, median standard error. “bias”, bias in correlation coefficient R^2^. “empse”, empirical standard error. “mse”, mean squared error. “relprec”, percentage gain in precision relative to the reference method. “modelse”, model-based standard error. “relerror”, relative percentage error in standard error. “cover”, coverage of a nominal level. “becover”, bias-eliminated coverage of a nominal level. “power”, power of a (1 - level) (defaults to NULL, in which case all summary statistics are returned). “mcse”, Mont Carlo standard errors. “lower” and “upper” denote the lower limit and upper limit of the Mont Carlo 95% confidence intervals.

(See separate excel file)

## Table S5. Summary statistics for the correlation coefficient (R^2^) between the simulated exposure and PRS in the simulation analysis investigating the impact of the number of SNPs involved in GWAS for genetic instrument discovery on results.

The correlation coefficient R^2^ is noted as theta in the table. “nsim”, the number of repeated simulation. “thetamean”, mean of correlation coefficient R^2^. “thetamedian”, median of correlation coefficient R^2^. “bias”, bias in correlation coefficient R^2^. “empse”, empirical standard error. “mse”, mean squared error. “relprec”, percentage gain in precision relative to the reference method. “mcse”, Mont Carlo standard errors. “lower” and “upper” denote the lower limit and upper limit of the Mont Carlo 95% confidence intervals respectively.

(See separate excel file)

## Table S6. Summary statistics for the two-stage least squares effect estimate (beta coefficient) of the exposure on the outcome in the simulation analysis investigating the impact of the number of SNPs involved in GWAS for genetic instrument discovery on results.

The beta coefficient is noted as theta in the table. “nsim”, the number of repeated simulation. “thetamean”, mean of correlation coefficient R^2^. “thetamedian”, median of correlation coefficient R^2^. “se2mean”, average standard error. “se2median”, median standard error. “bias”, bias in correlation coefficient R^2^. “empse”, empirical standard error. “mse”, mean squared error. “relprec”, percentage gain in precision relative to the reference method. “modelse”, model-based standard error. “relerror”, relative percentage error in standard error. “cover”, coverage of a nominal level. “becover”, bias-eliminated coverage of a nominal level. “power”, power of a (1 - level) (defaults to NULL, in which case all summary statistics are returned). “mcse”, Mont Carlo standard errors. “lower” and “upper” denote the lower limit and upper limit of the Mont Carlo 95% confidence intervals.

(See separate excel file)

## Table S7. Summary statistics for the correlation coefficient (R^2^) between the simulated exposure and PRS in the simulation analysis investigating the impact of the number of jackknife blocks involved in GWAS for genetic instrument discovery on results.

The correlation coefficient R^2^ is noted as theta in the table. “nsim”, the number of repeated simulation. “thetamean”, mean of correlation coefficient R^2^. “thetamedian”, median of correlation coefficient R^2^. “bias”, bias in correlation coefficient R^2^. “empse”, empirical standard error. “mse”, mean squared error. “relprec”, percentage gain in precision relative to the reference method. “mcse”, Mont Carlo standard errors. “lower” and “upper” denote the lower limit and upper limit of the Mont Carlo 95% confidence intervals respectively.

(See separate excel file)

## Table S8. Summary statistics for the two-stage least squares effect estimate (beta coefficient) of the exposure on the outcome in the simulation analysis investigating the impact of the number of jackknife blocks involved in GWAS for genetic instrument discovery on results.

The beta coefficient is noted as theta in the table. “nsim”, the number of repeated simulation. “thetamean”, mean of correlation coefficient R^2^. “thetamedian”, median of correlation coefficient R^2^. “se2mean”, average standard error. “se2median”, median standard error. “bias”, bias in correlation coefficient R^2^. “empse”, empirical standard error. “mse”, mean squared error. “relprec”, percentage gain in precision relative to the reference method. “modelse”, model-based standard error. “relerror”, relative percentage error in standard error. “cover”, coverage of a nominal level. “becover”, bias-eliminated coverage of a nominal level. “power”, power of a (1 - level) (defaults to NULL, in which case all summary statistics are returned). “mcse”, Mont Carlo standard errors. “lower” and “upper” denote the lower limit and upper limit of the Mont Carlo 95% confidence intervals.

(See separate excel file)

## Table S9. Results of one-sample MR on the effect of body mass index (BMI) on circulating blood biomarkers in the UK Biobank, where BMI were instrumented by weighted PRS constructed by three different GWAS frameworks.

External (GIANT) refers to the results generated using externally weighted BMI PRS constructed with Locke et al. GWAS summary statistics. Jackknife refers to the results generated using block jackknife resampling BMI PRS constructed with UK Biobank (UKB) data. Overlap refers to the results generated using internally weighted BMI PRS constructed from a GWAS of overlapping samples for UKB participants.

(See separate excel file)

## Table S10. Results from block jackknife resampling MR on the effects of childhood and adult body size on the levels of testosterone in adulthood.

Table S10A. Block jackknife resampling MR results on the effects from childhood and adult body size on the levels of testosterone in adulthood in all participants and sex-stratified datasets (univariable model).

Table S10B. Block jackknife resampling MR results on the effects from childhood and adult body size on the levels of testosterone in adulthood in males (multivariable model).

Table S10C. MR results on the effects from the levels of testosterone in adulthood on childhood and adult body size in males (univariable model), generated using block jackknife resampled PRS ("Jackknife") or PRS constructed with GWAS on overlapping samples ("Overfit").

(See separate excel file)

# References

1. Burgess S, Small DS, Thompson SG. A review of instrumental variable estimators for Mendelian randomization. Stat Methods Med Res. 2017;26(5):2333-55.

2. Allen NE, Sudlow C, Peakman T, Collins R, Biobank UK. UK biobank data: come and get it. Sci Transl Med. 2014;6(224):224ed4.

3. Collins R. What makes UK Biobank special? The Lancet. 2012;379(9822):1173-4.

4. Bycroft C, Freeman C, Petkova D, Band G, Elliott LT, Sharp K, et al. The UK Biobank resource with deep phenotyping and genomic data. Nature. 2018;562(7726):203-9.

5. O'Connell J, Sharp K, Shrine N, Wain L, Hall I, Tobin M, et al. Haplotype estimation for biobank-scale data sets. Nat Genet. 2016;48(7):817-20.

6. Huang J, Howie B, McCarthy S, Memari Y, Walter K, Min JL, et al. Improved imputation of low-frequency and rare variants using the UK10K haplotype reference panel. Nat Commun. 2015;6:8111.

7. Howie B, Marchini J, Stephens M. Genotype imputation with thousands of genomes. G3 (Bethesda). 2011;1(6):457-70.

8. Mitchell R, Hemani G, Dudding T, Paternoster L. UK Biobank Genetic Data: MRC-IEU Quality Control, Version 2. data.bris; 2018.

9. Loh PR, Tucker G, Bulik-Sullivan BK, Vilhjalmsson BJ, Finucane HK, Salem RM, et al. Efficient Bayesian mixed-model analysis increases association power in large cohorts. Nat Genet. 2015;47(3):284-90.
